# Fast Response and Spontaneous Alignment in Liquid Crystals Doped with 12-Hydroxystearic Acid Gelators

**DOI:** 10.3390/ma11050745

**Published:** 2018-05-07

**Authors:** Hui-Chi Lin, Chih-Hung Wang, Jyun-Kai Wang, Sheng-Feng Tsai

**Affiliations:** Department of Electro-Optical Engineering, National Formosa University, Yunlin 632, Taiwan; power99832@yahoo.com.tw (C.-H.W.); bruce830509@gmail.com (J.-K.W.); gentle0314@hotmail.com (S.-F.T.)

**Keywords:** liquid crystal, alignment, gelator, fast reponse, multistable property

## Abstract

The spontaneous vertical alignment of liquid crystals (LCs) in gelator (12-hydroxystearic acid)-doped LC cells was studied. Gelator-induced alignment can be used in both positive and negative LC cells. The electro-optical characteristics of the gelator-doped negative LC cell were similar to those of an LC cell that contained a vertically aligned (VA) host. The rise time of the gelator-doped LC cell was two orders of magnitude shorter than that of the VA host LC cell. The experimental results indicate that the gelator-induced vertical alignment of LC molecules occurred not only on the surface of the indium tin oxide (ITO) but also on the homogeneous alignment layer. Various LC alignments (planar, hybrid, multistable hybrid, and vertical alignments) were achieved by modulating the doped gelator concentrations. The multistable characteristic of LCs doped with the gelator is also presented. The alignment by doping with a gelator reduces the manufacturing costs and provides a means of fabricating fast-responding, flexible LC displays using a low-temperature process.

## 1. Introduction

The alignment of liquid crystals (LCs) is very important for LC devices such as sensors [1,2,3,4], light shutters [5,6,7], and displays [8,9], because it causes them to exhibit a specific uniform orientation. LCs can be aligned using several methods; in the liquid crystal display (LCD) industry, the molecular orientation of LCs is mainly achieved by rubbing polyimide films. Photoalignment is a contact-free alignment technique that involves photodegradation, photo-crosslinking, and photoisomerization [10,11,12,13]. Photoalignment provides an easy means of patterning and avoids the formation of dust and electrostatic charges. Langmuir–Blodgett films that are formed by mixing fatty acids with LCs can cause LC molecules to be aligned perpendicularly to substrate surfaces [14,15]. However, Langmuir–Blodgett films need to be deposited under a fixed surface pressure and then be transferred onto substrates at a different surface pressure. Nanoporous anodic aluminum oxide films [16,17], zinc oxide nanorods, and nanowires [18] grown vertically on indium tin oxide (ITO) layers have been used to align LCs. These inorganic materials can tolerate high temperatures and provide high reliability. Doping different types of nanoparticles (NPs), such as polyhedral oligomeric silsesquioxane (POSS) [19,20], alumina NPs [21], metallic NPs [22,23,24], and ionic surfactant [25] into an LC film can induce the microscopic alignment of LC molecules. Achieving LC alignment by dispersing NPs into LCs is an easier method than using conventional polymer layers. In recent researches, gelator-doped LCs have shown to possess the multistable characteristic, and some devices, such as phase gratings and phase-retardation plates based on gelator-doped LCs have also been realized [26,27]. In this investigation, the spontaneous vertical alignment of gelator-doped LC cells was examined. The electro-optical characteristics of the gelator-doped LCs were evaluated and compared with those of a vertically aligned (VA) host LC cell. The multistable property and various alignments in gelator-doped LCs are also presented.

## 2. Materials and Methods

**Figure 1:**
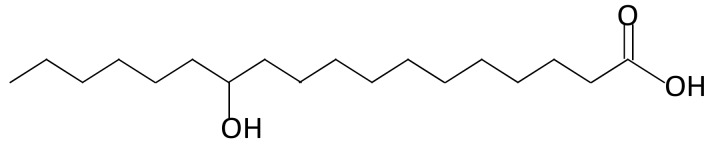
Chemical structure of 12-hydroxystearic acid (12-HSA).

In the experiments, all materials were purchased from commercial suppliers and used without further purification. Both positive and negative dielectric anisotropic LCs were used. The positive dielectric anisotropic LCs used were HFW59200-200 [26,27,28] (clearing temperature *T*_c_ = 113 °C, FUSOL MATERIAL) and E7 [29,30,31] (*T*_c_ = 58 °C, ECHO CHEMICAL). The birefringence (Δ*n*) at 589 nm and dielectric constant anisotropy (Δ*ε*) at 1 kHz of HFW59200-200 are 0.107 and 9.5, respectively, while E7 has Δ*n* = 0.2246 and Δ*ε* = 13.8. The negative dielectric LC (LCT-06441) used possesses Δ*n* = 0.102 and Δ*ε* = −4, with *T*_c_ = 86.8 °C. The rotational viscosity (*γ*) and elastic constants (*K*_1_ and *K*_3_) of LCT-06441 at 20 °C are *γ* = 210, *K*_1_ = 15.1, and *K*_3_ = 14.6, respectively. All LCs were in nematic state at room temperature. The doping material utilized was 12-hydroxystearic acid (12-HSA) [26,27,32], which can be produced by hydrogenation of the double bond of castor oil and is an organic gelator with a low molecular weight. The chemical structure of 12-HSA is shown in Figure 1.

First, the gelator was dissolved in each LC by increasing the temperature to 100 °C. The resulting homogeneous gelator/LC mixture was injected into an empty cell at 100 °C, controlled by a heating stage (LINKAM LTS 120E). The empty cells were composed of two ITO-coated glass substrates without alignment treatments to test the spontaneous vertical alignment of the LCs. The sample was cooled at 10 °C/min to room temperature. For comparison, a VA-negative LC cell was fabricated. The cleaned glass slides were immersed in an aqueous solution containing 1.5% (*w*/*v*) *N*,*N*-dimethyl-*N*-octadecyl-3-aminopropyltrimethoxysilyl chloride (DMOAP, Sigma-Aldrich, St. Louis, MO, USA) (Changed.) for 20 min and then rinsed with deionized water for 5 min. The DMOAP-coated glass slides were dried under a stream of nitrogen and baked at 100 °C for 1 h to generate homeotropic alignment films. The cell gap of the empty cell without alignment treatments or with VA was ~3.4 µm. To improve the uniformity of the bright state of the samples, gelator-doped and VA-negative LC cells with the rubbing treatment were also fabricated. For the gelator-doped LC cell, ITO glass substrates were treated by antiparallel rubbing before being assembled into a cell, while the rubbing treatment was performed on the DMOAP films in the VA LC cell. These samples were rubbed using a rayon-cloth rubbing machine at a rotation speed of 300 rpm, a plate speed of 10 mm/s, and a pile impression of 0.5 mm for four times.

For the test of the electronically controlled birefringence of positive LCs doped with gelator, one ITO substrate of the cell was coated with a planar alignment layer, and the other had no alignment treatments. The cleaned glass slide was immersed in 0.1 wt % solutions of polyvinyl alcohol (PVA, Sigma-Aldrich) in water for 3 min and baked at 120 °C for 20 min. The PVA film on the substrate was rubbed to generate a homogeneous alignment film. The 12-HSA/HFW59200-200 mixture was injected into the empty cell with a cell gap of ~3.6 µm by means of the same method as mentioned above.

## 3. Results and Discussion

### 3.1. Spontaneous Vertical Alignment in LCs Doped with Gelator

**Figure 2:**
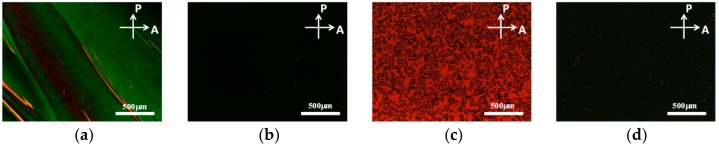
Polarized optical microscopy (POM) images of (**a**) pure liquid crystals (LC) HFW59200-200; (**b**) 0.1 wt %-12-HSA-doped HFW59200-200; (**c**) pure LC E7; and (**d**) 0.1 wt %-12-HSA-doped E7 cells, obtained between crossed polarizers.

Figure 2 shows polarized optical microscopy (POM) images of pure LC HFW59200-200, 0.1 wt %-12-HSA-doped HFW59200-200, pure LC E7, and 0.1 wt %-12-HSA-doped E7 cells without alignment treatment. The two undoped samples appear as inhomogeneous green and red images, respectively, between crossed polarizers (Figure 2a,c), whereas the images of the doped samples are dark, as displayed in Figure 2b,d. The multi-domain orientations of the LC molecules and the phase retardation of the sample are responsible for the inhomogeneous colors in Figure 2a,c. When 0.1 wt % 12-HSA was doped into HFW59200-200 and E7, dark images were obtained because of the homeotropic orientation of the LC molecules. The reason for the vertical orientation of the LC molecules is as follows. When each gelator/LC mixture was cooled at 10 °C/min from a high temperature (100 °C), the gelator molecules and the LC medium underwent phase separation. The gelator molecules slowly diffused toward the solid ITO surfaces. Since the hydrophilic carboxylic acid at the end of each 12-HSA molecule interacts with the ITO solid substrate much more strongly than its hydrophobic alkyl chain, the 12-HSA molecules spontaneously assemble on the ITO substrates with their molecular orientation normal to the ITO surface [33]. These self-assembled 12-HSA molecules on the substrates function as vertical alignment materials, causing the LC molecules to orientate homeotropically.

**Figure 3:**
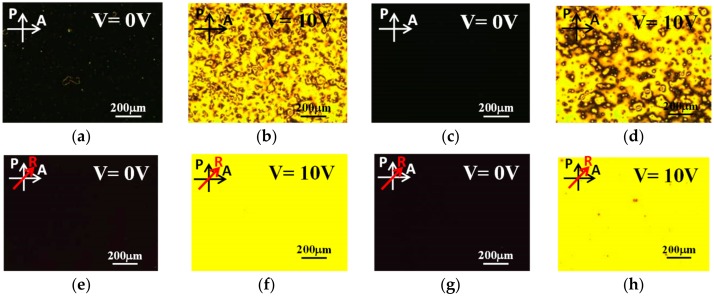
POM images of (**a**,**b**,**e**,**f**) 0.1 wt %-gelator-doped negative dielectric LC LCT-06441 and (**c**,**d**,**g**,**h**) VA host LC cells; (**a**–**d**) and (**e**–**h**) are images of samples without and with rubbing, respectively.

Figure 3a presents a POM image of a 0.1 wt %-gelator-doped negative-dielectric LC (LCT-06441) cell without alignment layers between crossed polarizers. As above, the image is dark because the self-assembled gelator molecules induce the vertical orientation of the LCs. Small bright spots and circles are observed owing to the weak alignment of the negative LC molecules by 0.1 wt % gelator. Applying an external voltage of 10 V to the cell reoriented the LC molecules perpendicular to the electric field, resulting in a bright state, as shown in Figure 3b. Many disclination lines appeared because the LC molecules tilted in various azimuthal directions under the applied voltage. For comparison, a VA-negative LC cell was fabricated. Homeotropic alignment was achieved by coating ITO glass slides with the surfactant DMOAP. Figure 3c,d display the dark and bright states of the VA host LC cell, respectively. The results are similar to those obtained for the gelator-doped negative LC cell. The water contact angles of the DMOAP and the self-assembled gelator alignment layers were measured to be 97.81° and 89.59°, respectively. The contact angles of the two films were high, conforming to the characteristic of vertical alignment layers.

The disclination lines in Figure 3b,d reduced the transmission of the bright state and the contrast ratio of the sample. To improve the uniformity of the bright state of the samples, gelator-doped and VA-negative LC cells with the rubbing treatment were fabricated. ITO glass substrates were treated by antiparallel rubbing before being incorporated into the gelator-doped LC cell, while rubbing was performed on the DMOAP films in the VA LC cell. A more uniform dark state at *V* = 0 (Figure 3e) and a more uniform bright state at *V* = 10 V (Figure 3f) were observed in the rubbed doped LC cell than in the original cell. The reason is that the rubbing treatment brings many grooves on ITO films. When the gelator/LC mixture was injected into the empty cell with ITO substrates covered with grooves, a few LC molecules lay into the grooves and oriented parallel to the rubbing direction. The grooves provided a weak homogeneous alignment effect for the LC molecules. The effect of the grooves and the vertical-alignment effect of 12-HSA combined to result in a small change of the LC pretilt angle, which caused a uniform reorientation of the LC molecules. The rubbing treatment also improved the contrast ratio of the VA host LC cells (Figure 3g,h) because the homeotropically aligned LC molecules tilted in the rubbing direction when the applied voltage exceeded a threshold value.

### 3.2. Electro-Optical Properties of Gelator-Doped Negative LC Displays

**Figure 4:**
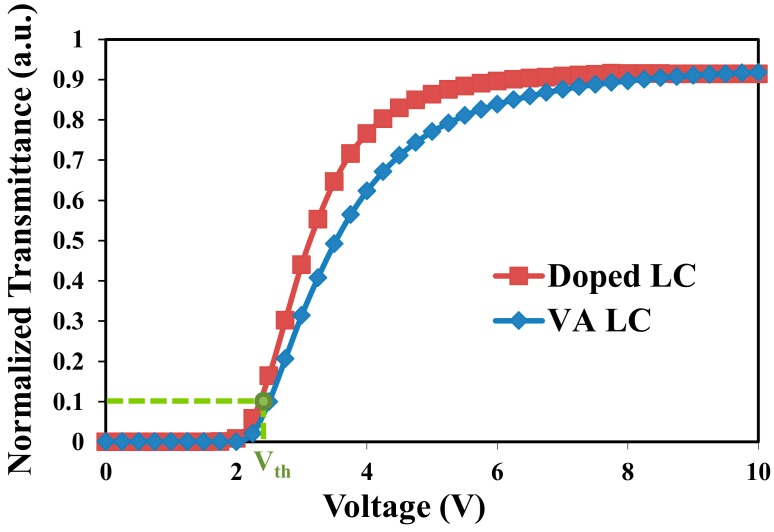
Electro-optical curves of 0.1 wt %-gelator-doped negative LC LCT-06441 (red squares) and VA host LC (blue diamonds) cells.

**Figure 5:**
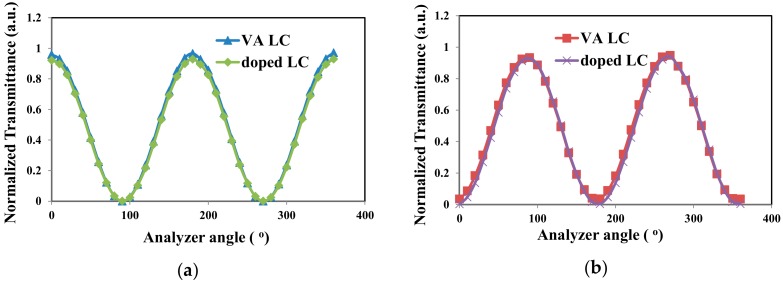
Transmission of 0.1 wt %-gelator-doped LCT-06441 and VA host LC cells positioned between two polarizers, with the analyzer axis rotated relative to the polarizer axis. Angular dependence of the transmission under applied voltages of (**a**) *V* = 0 V and (**b**) *V* = 10 V.

Figure 4 plots the electro-optical curves of 0.1 wt %-gelator-doped negative LC and VA host LC cells with rubbing. The measured cell gaps in the two samples were ~3.4 µm. The two curves in Figure 4 are similar. The threshold voltages of the two samples were both ~2.4 V. The contrast ratios of the doped LC and VA host LC cells, measured from Figure 4, were 2107 and 1311, respectively. The variations of the transmission of gelator-doped negative LC are plotted in Figure 5a,b, and VA host LC cells are shown in Figure 3e–h, respectively, with the analyzer angle. The angle of the analyzer is between the transmission axes of the two polarizers. Figure 5a represents the results obtained for the two samples without an applied voltage. The two curves are almost identical, indicating that the LC molecules in the two samples are aligned vertically and exhibit no phase retardation. When a voltage of 10 V was applied to the samples, the polarization of the output laser light changed, as shown in Figure 5b. The two curves in Figure 5b are very similar and reveal a phase retardation of ~π. These results demonstrate that the ability of 12-HSA to align vertically the LC molecules is comparable with that of the conventional alignment material DMOAP. The durability of the gelator-doped LC cell upon the repeated application of the voltage was also measured. The transmissions of the cell were almost the same after the voltage was switched on (10 V) and off 100 times.

The rise and decay times of a homeotropic LC cell depend on the parameters viscosity (*γ*), cell gap (*d*), and *V* as follows [34].

(1) $$\tau_{rise}=\frac{\gamma d^{2}}{K_{33}\pi^{2}\left[(V/V_{th}{)}^{2}-1\right]},$$

(2) $$\tau_{decay}=\frac{\gamma d^{2}}{K_{33}\pi^{2}\left[(V_{b}/V_{th}{)}^{2}-1\right]},$$

where *K_33_* is the elastic constant, *V_th_* and *V_b_* are threshold and bias voltages, respectively. When *V* was 10 V (frequency *f* = 1 kHz), the rise and decay times of the 0.1 wt %-gelator-doped negative LC cell were 49.6 and 15.6 ms, respectively. The rise and decay times are defined as the time needed for the transmittance to change from 10% to 90% and from 90% to 10%, respectively [35,36]. The VA-negative LC cell had a similar decay time to that of the doped LC cell, but a longer rise time (247 ms) than the doped cell. The long rise times of the two samples were caused by the optical bounce that appeared at the rising edge of the optical response curve. The doped LC cell had a faster rise time than the VA LC cell because cyclic dimers of the carboxylic acid group formed between the gelator molecules dispersed in the LC bulk [32]. The aggregated gelators functioned as the polymer sticks in polymer-stabilized LC (PSLC) cells [37,38,39], suppressing the optical bounce and improving the rise time of the cell.

**Figure 6:**
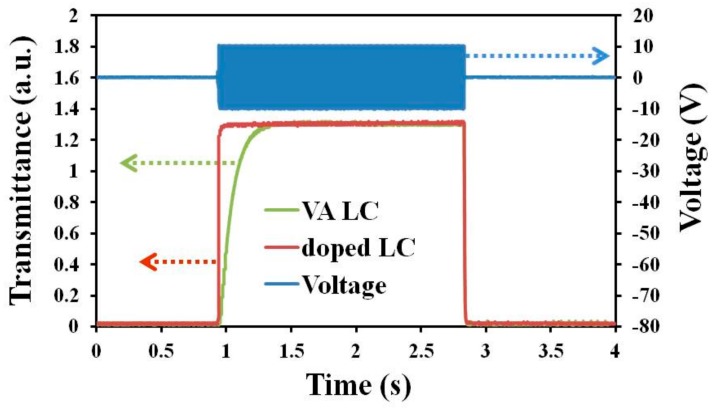
Optical transmittance of 0.1 wt %-gelator-doped LCT-06441 and VA host LC cells under a step-voltage driving scheme with a step maintenance time of 3 ms.

To shorten the slow rise time of the samples, a step-voltage driving scheme was applied, by gradually increasing *V* from 0 to 10 V in steps of 2 V. Each voltage step was maintained for 3 ms, and *V* was then relaxed to 0 V directly from 10 V to measure the decay time. When a voltage is applied to an LC cell, the reorientation of the LC molecules in the bulk brings LC flow, which causes a bounce in the optical transmittance curve and induces a slow response time. A lower voltage leads to a smaller/no optical bounce [37]. Therefore, the step-voltage driving scheme was used to decrease the rise time of the LCs. Figure 6 displays the optical transmittance of the 0.1 wt %-gelator-doped and VA-negative LC cells under the step-voltage driving scheme. The optical bounces of the two samples disappeared completely under step-voltage driving. A rise time of 4.72 ms and a decay time of 6.72 ms were obtained for the doped LC cell. The VA LC cell had a rise time of 193 ms and a decay time of 7.04 ms. These results revealed that the step-voltage driving scheme improved not only the rise time but also the decay time, because the scheme reduced the effect of LC flow. The response time (rise time plus decay time) of the doped LC cell that was obtained using the step-voltage driving scheme was just ~18% of that obtained using the conventional pulse voltage method. The rise time of the doped LC cell was only ~2.4% of that of the VA host LC cell when the step-voltage driving scheme was used. These results indicate that the gelator-doped LC cell was two orders of magnitude faster than the VA host LC cell when the step-voltage driving scheme was applied (Table 1). The step-voltage driving scheme was more effective for the doped LC cell than for the VA LC cell because the step-voltage maintenance time (3 ms) matched the rise time of the doped LC cell.

When the maintenance time of each voltage step in the step-voltage driving scheme increased to 5 ms, the rise time of the doped LC cell was slightly lengthened, and the rise time of the VA LC cell was shortened, as presented in Table 1, because the step-voltage maintenance time had to match the reorientation time of the LC cell. Therefore, when the maintenance time was extended to 10 ms, the rise time of the VA LC cell was reduced to 108 ms, which is 56% of that obtained using the step-voltage driving scheme with a maintenance time of 3 ms. The rise time of the VA LC cell can be improved further by modulating the maintenance time of the voltage step; however, it still will not compete with that of the gelator-doped LC cell.

### 3.3. Various Alignments and Multistable Property of Gelator-Doped Positive LCs

**Figure 7:**
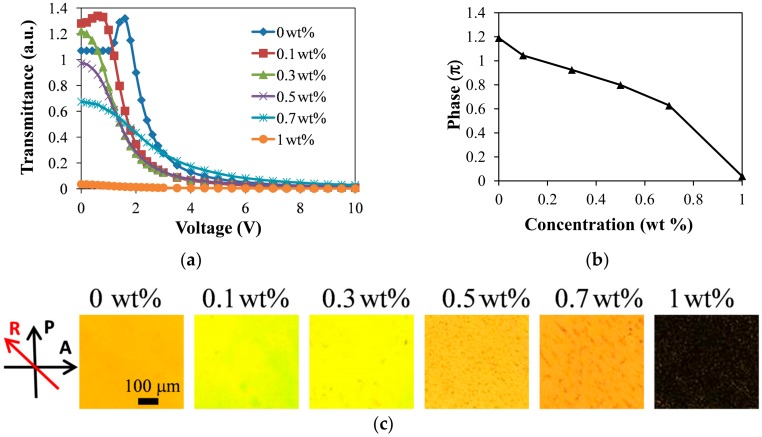
(**a**) Electro-optical curves; (**b**) phases; and (**c**) POM images of positive LCs (HFW59200-200) doped with various gelator concentrations.

**Figure 8:**
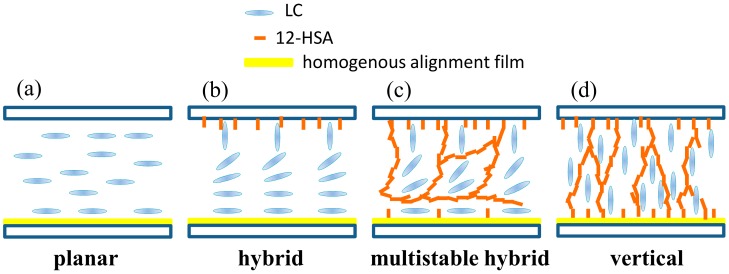
Schematics of LC orientations after doping with the gelator. (**a**) Planar, (**b**) hybrid, (**c**) multistable hybrid, (**d**) vertical orientations.

In this part of the experiment, the multistable property and various alignments of gelator-doped positive LC (HFW59200-200) cells were observed. In the LC cell, one substrate was coated with a PVA homogenous-alignment film and rubbed, whereas in the other substrate, no alignment treatment was performed. Figure 7 shows the electro-optical curves, the phase retardations, and the POM images of the positive LC that was doped with various gelator concentrations. The results indicate that the phase retardations varied with the concentration of dopant. The curve of the undoped LC cell in Figure 7a has a peak and reveals a threshold voltage of 1.2 V. The undoped LC cell with no bias exhibited a phase retardation of ~1.2π, as shown in Figure 7b. The orientation of LC molecules in the undoped cell with only one alignment layer (depicted in Figure 8a) was similar to that of homogenously aligned LC cells, because of the elastic continuum theory. When the gelator concentration was 0.1 wt %, the LC molecules near the bare ITO surface were aligned vertically because the gelator molecules self-assembled on the ITO substrates with their molecular orientation normal to the ITO surface, as shown in Figure 8b. Consequently, the phase retardation reduced, and the threshold voltage disappeared (red square-dot curve in Figure 7a), because not all LC molecules were parallel to the substrate. The results demonstrate that the phase of the sample decreased to less than π, and the peak of the electro-optical curve vanished as the gelator concentration reached 0.3 wt %. When the concentration of dopant was 0.7 wt %, the phase retardation was ~0.6π—close to that of a hybrid aligned nematic LC cell (Figure 8c). When the gelator concentration was sufficiently high (1 wt %), the electro-optical curve was almost a straight line, the phase retardation was close to zero, and the POM image was dark (Figure 7c). The result was due to the vertical alignment of the LC molecules by the gelator, not only on the surface of ITO but also on the PVA homogenous alignment layer, as shown in Figure 8d. In the LC cell, a hydrogen bond formed between the hydrophilic carboxylic acid of 12-HSA and the hydroxyl of PVA. 12-HSA on the PVA surface produced a similar effect to that on the ITO surface, providing a vertical anchoring structure for LC molecules. A higher dopant concentration gave rise to a higher tilt angle of the LC molecules. The LC molecules in the 1 wt %-gelator-doped LC cell were oriented almost vertically owing to the sufficiently high 12-HSA concentration. If doping materials possessing amine or hydroxyl groups are used, the phenomenon of vertical alignment for LC molecules may weaken because of a weak hydrogen bond between the hydroxyl of PVA and the amine/hydroxyl group of the doping materials.

**Figure 9:**
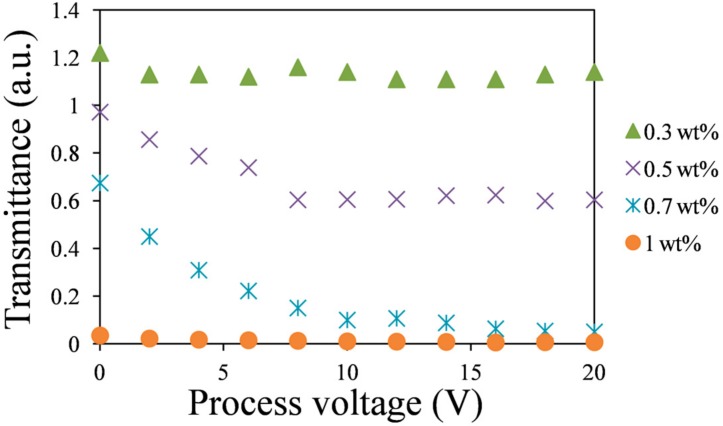
Multistable *T–V* curves of HFW59200-200 cells doped with various gelator concentrations. The transmittance was measured without an applied voltage.

Figure 9 plots the measured multistable characteristic of the positive LC cells that were doped with various concentrations of gelator. The sample in the multistable measurement was initially heated to 100 °C. Its temperature was held at 100 °C, and an external voltage was applied to it. Thereafter, the sample was cooled at 10 °C/min to room temperature, as an external electric field was applied. The external field was then turned off. This applied voltage in the multistable measurement was called process voltage and was changed from zero to 20 V. Figure 9 represents multistable transmittance–voltage (*T–V*) curves of HFW59200-200 LC cells that were doped with various gelator concentrations. Each transmission in Figure 9 was measured without the application of voltages. The results revealed few variations of transmittance in the 0.3 wt %-gelator-doped LC cell with process voltage because this mixed LC material remained mobile. Experimentally, the LCs that was doped with 0.5 wt % or more gelator formed gels at room temperature owing to hydrogen bonding between the gelator molecules. The curve of the 0.5 wt %-gelator-doped LC cell demonstrates a limited multistable effect. The gelator molecules can fix LC orientations at low tilt angles by thermoreversible association and dissociation. Liquid crystal molecules at higher tilt angles cannot be stabilized because the dopant concentration is lower. At a dopant concentration of 0.7 wt %, various transmissions at process voltages could be maintained, and the *T–V* curve was similar to that obtained when a voltage was applied (the blue asterisk curve in Figure 7a). In this cell, the gelator molecules automatically assembled in the direction of the reoriented LCs and stabilized the LC molecules at any tilt angles under the process voltage, achieving multistable LC orientations [26,27]. When the gelator concentration was high (1 wt %), the LC molecules oriented almost vertically, as shown in Figure 8d. The multistable characteristic of the cell could be observed, but the change of transmittance was very weak.

## 4. Conclusions

In conclusion, this study investigated the spontaneous induction of the vertical alignment of LC cells by a gelator. The electro-optical curve of a gelator-doped negative LC cell was similar to that of a VA host LC cell. The doped LC cell had a faster response time than a VA host LC cell. The rise time of the doped LC cell was ~2.4% of that of the VA host LC cell when a step-voltage driving scheme with a step maintenance time of 3 ms was used. Furthermore, the results indicate that the gelator induced the vertical alignment of the LC molecules not only on the ITO but also on PVA homogeneous-alignment surfaces. Various LC structures were achieved by modulating the gelator concentrations. Tunable phase retardation and multistable characteristics of the doped LC cell were demonstrated.

## Figures and Tables

**Table 1:** Rise times of 0.1 wt %-gelator-doped and VA LCT-06441 cells under a step-voltage driving scheme with various step-voltage maintenance times.

Maintenance Time (ms)	Rise Time (ms)	
	Doped LC	VA LC
3	4.72	193
5	6.96	115
10	–	108
